# The cost-effectiveness of using chronic kidney disease risk scores to screen for early-stage chronic kidney disease

**DOI:** 10.1186/s12882-017-0497-6

**Published:** 2017-03-13

**Authors:** Benjamin O. Yarnoff, Thomas J. Hoerger, Siobhan K. Simpson, Alyssa Leib, Nilka R. Burrows, Sundar S. Shrestha, Meda E. Pavkov, on behalf of the Centers for Disease Control and Prevention CKD Initiative

**Affiliations:** 1 RTI International, 3040 E. Cornwallis Road, P.O. Box 12194, Research Triangle Park, NC 27709-2194 USA; 2 Centers for Disease Control and Prevention, Atlanta, GA USA

**Keywords:** Chronic kidney disease, Risk scores, Screening

## Abstract

**Background:**

Better treatment during early stages of chronic kidney disease (CKD) may slow progression to end-stage renal disease and decrease associated complications and medical costs. Achieving early treatment of CKD is challenging, however, because a large fraction of persons with CKD are unaware of having this disease. Screening for CKD is one important method for increasing awareness. We examined the cost-effectiveness of identifying persons for early-stage CKD screening (i.e., screening for moderate albuminuria) using published CKD risk scores.

**Methods:**

We used the CKD Health Policy Model, a micro-simulation model, to simulate the cost-effectiveness of using CKD two published risk scores by Bang et al. and Kshirsagar et al. to identify persons in the US for CKD screening with testing for albuminuria. Alternative risk score thresholds were tested (0.20, 0.15, 0.10, 0.05, and 0.02) above which persons were assigned to receive screening at alternative intervals (1-, 2-, and 5-year) for follow-up screening if the first screening was negative. We examined incremental cost-effectiveness ratios (ICERs), incremental lifetime costs divided by incremental lifetime QALYs, relative to the next higher screening threshold to assess cost-effectiveness. Cost-effective scenarios were determined as those with ICERs less than $50,000 per QALY. Among the cost-effective scenarios, the optimal scenario was determined as the one that resulted in the highest lifetime QALYs.

**Results:**

ICERs ranged from $8,823 per QALY to $124,626 per QALY for the Bang et al. risk score and $6,342 per QALY to $405,861 per QALY for the Kshirsagar et al. risk score. The Bang et al. risk score with a threshold of 0.02 and 2-year follow-up screening was found to be optimal because it had an ICER less than $50,000 per QALY and resulted in the highest lifetime QALYs.

**Conclusions:**

This study indicates that using these CKD risk scores may allow clinicians to cost-effectively identify a broader population for CKD screening with testing for albuminuria and potentially detect people with CKD at earlier stages of the disease than current approaches of screening only persons with diabetes or hypertension.

## Background

Chronic kidney disease (CKD) affected 13.6% of U.S. adults in 2007–2012 [1] and is estimated to affect nearly 17% of adults by 2030 [2]. All stages of CKD have been shown to impose significant health and economic burden [3–5]. Better treatment during early stages of CKD may slow progression to end-stage renal disease (ESRD), the most severe stage of CKD, and reduce complications, medical costs, and mortality associated with CKD [6–9]. Achieving early treatment of CKD is challenging, however, because as many as 94.5% of persons with CKD are unaware of having the disease [10, 11]. Therefore, increasing awareness among patients and clinicians about CKD and CKD screening is important to achieve earlier treatment of CKD and mitigate its associated costs and complications. Screening for moderately increased albuminuria (microalbuminuria), a marker of early-stage CKD, was found to be cost-effective in populations with diabetes or hypertension [12–14]. Diabetes and hypertension are primary risk factors for CKD, but approximately 52% of those with CKD do not have diabetes, and approximately 10% do not have hypertension [1]. Thus, identifying cost-effective methods of screening for CKD in other populations is crucial to increase awareness of CKD and CKD screening among patients and clinicians and to improve early detection and management of CKD.

Using CKD risk scores to identify persons for CKD screening with testing for albuminuria may prove to be a cost-effective method for identifying a population broader than just those with diabetes or hypertension. In this study, we used the CKD Health Policy Model, a microsimulation model of CKD progression, to examine the cost-effectiveness of identifying persons for early-stage CKD screening (i.e., screening for moderate albuminuria) using two published CKD risk scores: one published by Bang et al. [15] and one published by Kshirsagar et al. [16]. We assessed the cost-effectiveness of alternative screening scenarios by varying risk score thresholds above which persons were assigned to receive screening and frequencies of follow-up screening if the initial test was negative.

## Methods

### Model overview

This study used the CKD Health Policy Model in 2015, a microsimulation model of CKD progression [2, 12, 17, 18]. Briefly, the model simulates progression of CKD and its complications in a nationally representative cohort drawn from the National Health and Nutrition Examination Survey (NHANES) through age 90 years or death. The model includes eight states: no CKD, CKD stages 1 through 5 (with stage 3 divided into 3a and 3b), and death. CKD stages are defined by estimated glomerular filtration rates (eGFR) and the presence of elevated albuminuria (urinary albumin to creatinine ratio ≥30 mg/g) [19]. The model concomitantly simulates the natural history of complications from CKD. Model parameters are derived from the epidemiological literature, clinical trials, and a previous cost-effectiveness study [14].

**Fig. 1:**
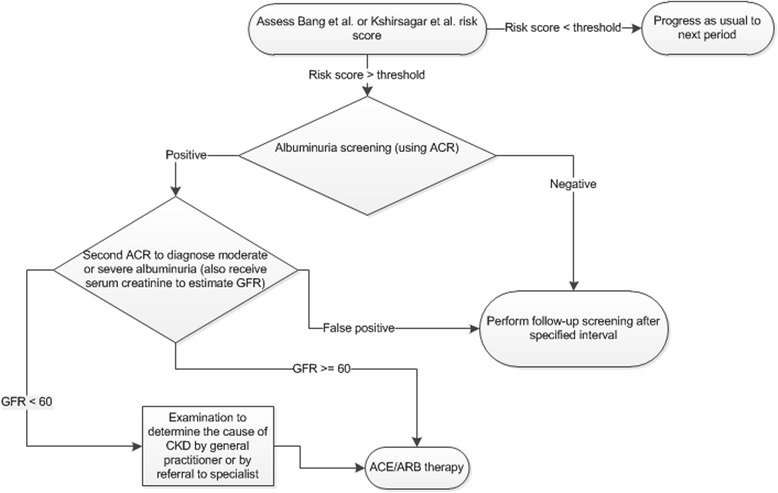
Flowchart of CKD screening and treatment in the CKD health policy model. ACE, angiotensin-converting enzyme inhibitor; ACR, albumin-to-creatinine ratio; ARB, angiotensin receptor blockers; CKD, chronic kidney disease; GFR, glomerular filtration rate

Importantly, the model simulates screening for moderate and severe albuminuria. Figure 1 presents the screening and treatment pathway in the model for a person diagnosed with CKD. In the model, treatment with angiotensin-converting enzyme (ACE) inhibitors or angiotensin receptor blockers (ARBs) decreases the probability of progression from moderate to severe albuminuria, slows the annual decline in GFR for persons with moderate albuminuria, and reduces the annual mortality rate for persons with moderate albuminuria. The model does not include parameters related to possible harms associated with screening, incidental findings, or over-diagnosis, but because of the two stage test and the sensitivity and specificity parameters, we expect misdiagnosis to be low. Model parameters related to CKD screening and treatments with ACE inhibitors or ARBs are shown in Table 1.

**Table 1:** Model Parameters for Albuminuria Screening, Treatment with Renin-angiotensin System Inhibitors, and Screening and Treatment Costs as Derived from Previous Publications

Model Parameter	Parameter Value	Source
Sensitivity of screening test for moderate albuminuria	0.73	Sarafidis et al. [23]
Specificity of screening test for moderate albuminuria	0.96	Sarafidis et al. [23]
Treatment adherence of persons diagnosed with moderate albuminuria	0.75	Boulware et al. [14]
Treatment effect relative risks (multiplied by baseline rates)		
Relative risk of moderate- to severe albuminuria transition for persons receiving treatment	0.45	Strippoli et al. [6]
Relative risk of mortality for persons with moderate albuminuria receiving treatment	0.77	Boulware et al. [14]
Relative risk of annual GFR decrease in persons with for persons with moderate albuminuria receiving treatment	0.67	Agodoa et al. [7] Ruggenenti et al. [8, 9]
Annual QALY decrement from CKD and related complications		
Proteinuria	0.01	Gorodetskaya et al. [24]
GFR 30–59	0.05	Gorodetskaya et al. [24]
GFR 15–29	0.07	Gorodetskaya et al. [24]
GFR <15	0.20	Gorodetskaya et al. [24]
Stroke, ever	0.582	Meenan et al. [25]
CA/MI, current year	0.12	Tsevat et al. [26]
CHD, ever without MI	0.053	Nease et al. [27]
Screening costs (2016 US $)		
Initial visit	88.58	CMS [28]
Second visit if positive during first visit	69.08	CMS [28]
One time costs of diagnostic tests to assess for CKD if GFR <60 ml/min/1.73 m^2^ (2010 $)		
Diabetes or hypertension	382.41	Boulware et al. [14, 20]; CMS [29]; AHRQ [30]
Neither hypertension nor diabetes: Severe albuminuria and age < 65	2,857.10	Boulware et al. [14, 20]; CMS [29]; AHRQ [30]
Neither hypertension nor diabetes: Moderate albuminuria and age <65	1,401.40	Boulware et al. [14, 20]; CMS [29]; AHRQ [30]
Neither hypertension nor diabetes: Age ≥65	964.69	Boulware et al. [14, 20]; CMS [29]; AHRQ [30]
Annual follow-up costs if GFR <60 ml/min/1.73 m^2^ (2016 US $)		
Specialist visit:		
Diabetes	94.15	Boulware et al. [14]; CMS [28]
No diabetes	85.23	Boulware et al. [14]; CMS [28]
General practitioner visit	132.88	Boulware et al. [14]; CMS [28]
Annual drug therapy		
ARBs (diabetes)	527.49	Boulware et al. [14]; Rodby et al .[31]; Lewis et al. [32]; Nakao et al. [33]; Jafar et al. [34]; Drug Topics Red Book [35]; AHRQ [36]
ACE inhibitors (no diabetes)	210.03	
Annual rate at which costs and QALYs are discounted (i.e. reduced)	3%	Weinstein et al. [21]

*GFR* glomerular filtration rate, *ACE* angiotensin converting enzyme inhibitor, *AHRQ* Agency for Healthcare Research and Quality, *ARB* angiotensin receptor blocker, *CKD* chronic kidney disease, *CMS* Centers for Medicare & Medicaid Services

### Costs

Costs in the model are lifetime costs (i.e. from model start to death) from the health care perspective. This includes all paid by insurers and paid out of pocket by patients (Table 1). The model does not include physician time for training in the use of the risk score although we expect that this may be minimal since the risk score can be implemented in existing EHRs. The model includes measures of costs for CKD screening. Initial costs include a physician visit to measure urine albumin and creatinine levels to identify the presence of moderate albuminuria and, if the test is positive, a second physician visit to confirm the presence of moderate albuminuria. Once the presence of moderate albuminuria has been confirmed, additional diagnostic costs are incurred if the person has eGFR less than 60 ml/min per 1.73 m^2^. The tests included in the one-time diagnosis costs are those identified in Boulware et al. [14, 20] as being most frequently recommended by primary care providers to test for CKD. Persons with moderate albuminuria also have annual treatment costs that include physician follow-up and either ACE inhibitors if they do not have diabetes or ARBs if they do. Costs are given in 2010 US dollars. To incorporate time preferences (i.e. persons prefer dollars and quality of life in the present to dollars and quality of life in the future), costs are discounted (i.e. reduced) by 3% annually as recommended for all cost-effectiveness analysis by Weinstein et al. [21].

### Risk scores

We assigned persons to receive CKD screening based on published risk scores. Risk scores were identified from literature review based on four criteria: (1) the predictive factors are commonly collected as part of regular physician office visits to ensure that the risk score can be feasibly implemented to identify a broad population for CKD screening, (2) the study pertains to the U.S. population, (3) the study has good internal predictive ability, and (4) the study has good external predictive ability as measured using external data sources. We allowed for the inclusion of some factors—diabetes, cholesterol, and anemia—that are collected at office visits with slightly less regularity.

Two risk scores were identified based on these criteria: one published by Bang et al. [15] and one published by Kshirsagar et al. [16] Bang et al. used logistic regression to predict current CKD (stage 3+) in the NHANES population and validated the prediction model in the Atherosclerosis Risk in Communities (ARIC) study. Kshirsagar et al. used logistic regression to predict onset of CKD (stage 3+) over the 9-year study period in subsamples of ARIC and the Cardiovascular Health Study (CHS) and validated the prediction model in other subsamples from ARIC and CHS.

The Bang et al. and Kshirsagar et al. risk scores range from zero to one and correspond to a person’s level of risk for having or developing CKD. Because the Bang et al. risk score is based on single observations per person in NHANES, it can be interpreted as the probability of a person having prevalent CKD. Because the Kshirsagar et al. risk score is based on longitudinal data from ARIC and CHS, it can be interpreted as the probability of developing CKD over 4 to 9 years of follow up. Clinically, the Bang et al. and Kshirsagar et al. risk scores can be used to evaluate a patient’s overall risk and determine whether the patient should receive CKD screening. Table 2 shows examples of the Bang et al. and Kshirsagar et al. risk scores for persons with different characteristics. The Bang et al. risk scores are generally lower for each group, except for those over age 70. The Bang et al. and Kshirsagar et al. risk scores are used only to identify persons at high risk of CKD who should receive screening. Coefficients from the logistic regressions for the two risk scores are shown in Table 3. These coefficients are derived from logistic regressions, so logistic transformation was used to construct risk scores in the model cohort, based on each person’s risk factors. The two risk scores are constructed using largely similar risk factors.

**Table 2:** Example Bang et al. and Kshirsagar et al. Risk Scores for Persons with Different Characteristics

Risk Score	No Risk Factors	Diabetes	Diabetes and Hypertension	Diabetes, Hypertension, and Anemia	Cardiovascular Disease
Bang et al.					
Female: Age < 50	0.01	0.01	0.01	0.04	0.06
Female: Age 50 to 59	0.03	0.04	0.06	0.15	0.24
Female: Age 60 to 69	0.06	0.09	0.13	0.27	0.40
Female: Age ≥ 70	0.13	0.19	0.27	0.49	0.63
Male: Age < 50	0.00	0.01	0.01	0.03	0.05
Male: Age 50 to 59	0.02	0.03	0.05	0.12	0.19
Male: Age 60 to 69	0.04	0.07	0.10	0.22	0.34
Male: Age ≥ 70	0.10	0.15	0.22	0.41	0.56
Kshirsagar et al.					
Female: Age < 50	0.04	0.06	0.09	0.14	0.18
Female: Age 50 to 59	0.07	0.10	0.16	0.24	0.28
Female: Age 60 to 69	0.14	0.18	0.28	0.38	0.45
Female: Age ≥ 70	0.15	0.20	0.30	0.41	0.48
Male: Age < 50	0.04	0.05	0.08	0.13	0.16
Male: Age 50 to 59	0.06	0.09	0.14	0.21	0.26
Male: Age 60 to 69	0.12	0.16	0.25	0.35	0.41
Male: Age ≥ 70	0.14	0.18	0.28	0.38	0.45

The Bang et al. risk score is derived from a logistic regression to predict current CKD (stage 3+) in the NHANES population. The Kshirsagar et al. risk score is derived from a logistic regression to predict onset of CKD (stage 3+) over the 9-year study period in subsamples of ARIC and the Cardiovascular Health Study (CHS). Coefficients used to generate the risk scores are given in Table 3

**Table 3:** Logistic Regression Coefficients by Individual Characteristics from Bang et al. and Kshirsagar et al. Risk Scores

Individual Characteristic	Bang et al. Risk Score Coefficient [15]	Kshirsagar et al. Risk Score Coefficient [16]
Age (Reference: 18 to 49)		
50 to 59	1.55	0.63
60 to 69	2.31	1.33
70+	3.23	1.46
Female	0.29	0.13
Diabetes	0.44	0.33
Hypertension^a^	0.45	0.55
Anemia^b^	0.93	0.48
Proteinuria^c^	0.83	-
History of cardiovascular disease	0.59	0.26
History of congestive heart failure	0.45	0.50
Constant	−5.40	−3.30

^a^Systolic blood pressure ≥ 140 mm/hg and/or diastolic blood pressure ≥ 90 mm/hg and/or use of antihypertensive medications

^b^Hemoglobin (Hb) concentration <12.0 g/dl

^c^Urinary Protein Excretion ≥ 30 mg/dl

After determining a person’s risk score, it was necessary to determine the risk score threshold over which a person is assigned to receive early-stage CKD screening (i.e., screening for moderate albuminuria). No optimal threshold was defined ex-ante, so five alternative thresholds (0.20, 0.15, 0.10, 0.05, and 0.02) were tested for the Bang et al. and Kshirsagar et al. risk scores. This range of thresholds was chosen because of the concentration of risk scores at the lower range of the distribution. For both risk scores, 95% of the cohort had a risk score less than 0.20. Persons with risk scores less than or equal to the threshold did not receive any screening or follow-up until their risk scores rose above the threshold. Once a person’s risk score rose above the risk score threshold, he or she was assigned to receive screening for moderate albuminuria. If the initial screening was negative, the person received a follow-up screening for moderate albuminuria at a specified interval. Because no optimal interval was defined ex-ante, we tested three intervals: 1 year, 2 years, and 5 years. These intervals were chosen because they have been used in past measures of CKD screening,[12] although there is no definitive recommendation for follow-up interval.

### Incremental cost-effectiveness ratios

Lifetime costs and quality-adjusted life years (QALYs) were simulated for the screening scenarios using the Bang et al. and Kshirsagar et al. risk scores described above for a nationally representative cohort drawn aged 30 or older from the 1999–2010 NHANES. The increment we evaluate is a change from the next highest risk score threshold, so each incremental change represents an increase in the number of people screened due to a lower risk score threshold. Lifetime costs and lifetime QALYs for each scenario were compared with the next higher risk score threshold to evaluate incremental costs and QALYs. The incremental cost-effectiveness ratios (ICERs) were computed as incremental lifetime cost divided by incremental lifetime QALYs for each screening scenario. Costs and QALYs were discounted (i.e. reduced) at a 3% annual rate, as recommended for all cost-effectiveness analysis by Weinstein et al. [21] We computed 95% confidence intervals for each ICER using a probabilistic sensitivity analysis where we allowed the following key model parameters to vary according to distributions taken from the literature: the hazard ratio of ACE inhibitor/ARB treatment on transition from moderate to severe albuminuria, the hazard ratio of ACE inhibitor/ARB treatment on eGFR decline, the hazard ratio of ACE inhibitor/ARB treatment on the annual mortality rate, ACE inhibitor inhibitor/ARB adherence, the costs of screening, and the costs of ACE inhibitor/ARB treatment.

Cost-effectiveness of any screening scenario depends on the specific willingness to pay for additional QALYs. The commonly used benchmark is $50,000 per QALY [22]. A screening scenario was determined to be cost-effective if the ICER per QALY gained is less than the willingness to pay threshold. The optimal scenario was determined as the cost-effective scenario that yields highest QALYs gained.

### Model validation

The external validity of the model was tested against data from the longitudinal ARIC study. The ARIC study tracked persons over approximately 9 years and included 4 office visits to collect laboratory data and health status information. Data from the first ARIC office visit were used to populate the simulation cohort in the model validation. We simulated 9 years in the model for this cohort and generated the distribution of the change in eGFR. The distribution of the actual change in eGFR between the first and last office visit in the ARIC study was compared with the simulated distribution to examine the performance of the model.

Results from validation testing of eGFR progression in the model demonstrated strong model performance. The model simulated a 9.72 mL/min per 1.73 m^2^ average decrease in eGFR over 9 years. In the ARIC data, the actual decrease in eGFR over the 9-year study period was 9.24 mL/min per 1.73 m^2^. The difference between the simulated and actual change in eGFR was statistically not different (i.e., *p* > 0.05).

### Sensitivity analysis

To test the sensitivity of our results and conclusions to the choice of parameters for risks and costs, we conducted a number of one-way sensitivity analyses by varying key model parameters by ±25%: the hazard ratio of ACE inhibitor/ARB treatment on transition from moderate to severe albuminuria, the hazard ratio of ACE inhibitor/ARB treatment on eGFR decline, the hazard ratio of ACE inhibitor/ARB treatment on the annual mortality rate, ACE inhibitor inhibitor/ARB adherence, the costs of screening, and the costs of ACE inhibitor/ARB treatment. We performed these tests for the optimal screening scenarios for each risk score identified in the main analysis. For each test, we examined the ICER relative to the no screening scenario and determined the percentage change from results in the main analysis. These parameters relate to the benefits and costs of early screening, so varying them tests the sensitivity of results to these benefits and costs. We also conducted probabilistic sensitivity analysis to generate 95% confidence intervals for simulation results.

## Results

Table 4 shows the cost-effectiveness of screening using the Bang et al. [15] and Kshirsagar et al. [16] risk scores for various risk score thresholds and screening follow-up frequencies. Using the Bang et al. risk score, lifetime QALYs and costs had only small differences across screening scenarios, but ICERs across the screening scenarios ranged from $8,823 per QALY to $124,626 per QALY. With annual follow-up screening, risk score thresholds of 0.10 or higher had ICERs below the willingness to pay benchmark of $50,000 per QALY. For both the 2-year and 5-year screening follow-up all risk score thresholds evaluated had ICERs less than the willingness to pay benchmark. Lower risk score thresholds had higher QALYs and in most cases also had higher ICERs than the next higher threshold. Among the cost-effective screening scenarios, a risk score threshold of 0.02 with 2-year follow-up had the highest lifetime QALYs (21.373) with an ICER of $19,116 per QALY.

**Table 4:** Cost-Effectiveness of Using the Bang et al. and Kshirsagar et al. Risk Scores to Identify Persons for CKD Screening

Screening Scenario	Means		ICER (2016 US $ per QALY)
	Lifetime Costs (2016 US $)	Lifetime QALYs	
No screening	$139,200	21.349	—
Bang et al. risk score			
1-year follow-up screening			
Risk threshold = 0.20	$139,945 (134,865, 147,248)	21.366 (20.659, 21.915)	$43,791 per QALY
Risk threshold = 0.15	$140,024 (135,020, 147,634)	21.367 (20.660, 21.903)	$79,408 per QALY
Risk threshold = 0.10	$140,152 (135,122, 147,313)	21.370 (20.660, 21.931)	$42,645 per QALY
Risk threshold = 0.05	$140,317 (134,909, 147,273)	21.372 (20.664, 21.919)	$82,165 per QALY
Risk threshold = 0.02	$140,566 (133,133, 145,052)	21.374 (20.602, 21.826)	$124,626 per QALY
2-year follow-up screening			
Risk threshold = 0.20	$139,783 (135,227, 147,465)	21.363 (20.688, 21.912)	$41,594 per QALY
Risk threshold = 0.15	$139,820 (135,201, 147,212)	21.365 (20.664, 21.888)	$18,749 per QALY
Risk threshold = 0.10	$139,880 (135,064, 147,565)	21.368 (20.652, 21.938)	$19,852 per QALY
Risk threshold = 0.05	$139,939 (134,902, 147,208)	21.370 (20.655, 21.904)	$29,778 per QALY
Risk threshold = 0.02	$139,997 (135,199, 147,283)	21.373 (20.666, 21.923)	$19,116 per QALY
5-year follow-up screening			
Risk threshold = 0.20	$139,708 (135,002, 147,573)	21.361 (20.686, 21.898)	$42,277 per QALY
Risk threshold = 0.15	$139,749 (135,108, 147,277)	21.363 (20.666, 21.890)	$20,403 per QALY
Risk threshold = 0.10	$139,775 (135,303, 147,168)	21.366 (20.649, 21.925)	$8,823 per QALY
Risk threshold = 0.05	$139,820 (134,826, 147,093)	21.368 (20.647, 21.917)	$22,609 per QALY
Risk threshold = 0.02	$139,852 (134,968, 147,288)	21.371 (20.644, 21.924)	$10,662 per QALY
Kshirsagar et al. risk score			
1-year follow-up screening			
Risk threshold = 0.20	$140,073 (135,007, 147,383)	21.366 (20.682, 21.915)	$51,316 per QALY
Risk threshold = 0.15	$140,179 (135,241, 147,683)	21.369 (20.664, 21.901)	$35,292 per QALY
Risk threshold = 0.10	$140,432 (135,483, 147,257)	21.371 (20.684, 21.916)	$126,832 per QALY
Risk threshold = 0.05	$140,657 (135,057, 147,402)	21.374 (20.648, 21.939)	$74,996 per QALY
Risk threshold = 0.02	$141,063 (135,111, 147,469)	21.375 (20.660, 21.946)	$405,861 per QALY
2-year follow-up screening			
Risk threshold = 0.20	$139,807 (134,840, 147,579)	21.363 (20.634, 21.916)	$43,328 per QALY
Risk threshold = 0.15	$139,848 (135,096, 147,115)	21.367 (20.658, 21.929)	$10,202 per QALY
Risk threshold = 0.10	$139,980 (135,162, 147,227)	21.370 (20.657, 21.899)	$44,115 per QALY
Risk threshold = 0.05	$140,022 (135,378, 147,369)	21.373 (20.708, 21.909)	$13,970 per QALY
Risk threshold = 0.02	$140,131 (134,999, 147,385)	21.374 (20.689, 21.918)	$109,186 per QALY
5-year follow-up screening			
Risk threshold = 0.20	$139,705 (135,092, 147,340)	21.361 (20.670, 21.893)	$42,001 per QALY
Risk threshold = 0.15	$139,744 (135,192, 147,309)	21.365 (20.659, 21.917)	$9,926 per QALY
Risk threshold = 0.10	$139,828 (135,188, 147,368)	21.367 (20.704, 21.887)	$41,910 per QALY
Risk threshold = 0.05	$139,853 (134,908, 147,298)	21.371 (20.651, 21.947)	$6,342 per QALY
Risk threshold = 0.02	$139,879 (134,675, 147,422)	21.372 (20.653, 21.925)	$25,366 per QALY

*CKD* chronic kidney disease, *ICER* incremental cost-effectiveness ratio, *QALYs* quality adjusted life years. Notes: 95% confidence intervals are in parentheses. Costs have been rounded to the nearest dollar, and QALYS have been rounded to the nearest thousandth. The Bang et al. risk score is derived from a logistic regression to predict current CKD (stage 3+) in the NHANES population. The Kshirsagar et al. risk score is derived from a logistic regression to predict onset of CKD (stage 3+) over the 9-year study period in subsamples of ARIC and the Cardiovascular Health Study (CHS). Coefficients used to generate the risk scores are given in Table 3

Using the Kshirsagar et al. risk score (Table 4), lifetime QALYs and costs had only small differences across screening scenarios, but ICERs across the screening scenarios ranged from $5,750 per QALY to $368,000 per QALY. With annual follow-up screening, risk score thresholds of 0.20 and 0.15 had ICERs lower than the willingness to pay benchmark. With 2-year follow-up, risk score thresholds 0.05 and higher had ICERs below the willingness to pay benchmark. With 5-year follow-up, all thresholds had ICERs lower than the willingness to pay benchmark. As with the Bang et al. risk score, using lower risk score thresholds had higher QALYs, however the pattern for ICERs was inconsistent. Among the cost-effective screening scenarios, a risk score threshold of 0.05 with 2-year follow-up screening had the highest lifetime QALYs (21.373) with an ICER of $12,667 per QALY. Comparing the optimal screening scenarios for the two risk scores, both yielded the same level of QALYs, but the optimal screening scenario using the Bang et al. score had a lower lifetime cost and therefore can be considered optimal overall.

**Fig. 2:**
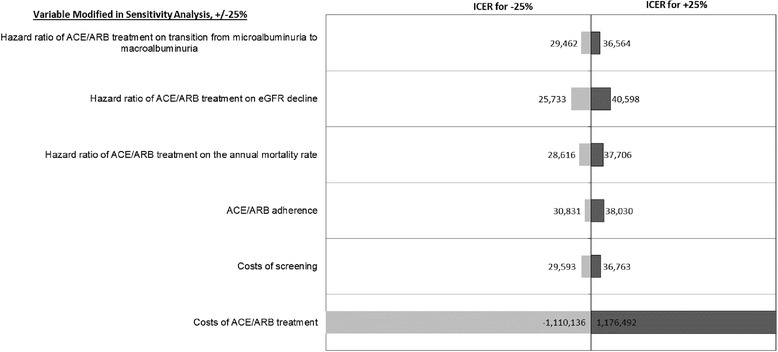
Sensitivity of results to 25% changes in specific parameters for screening and treatment, using a risk threshold of 0.02 for the Bang et al. risk score with 2-year follow-up screening relative to no screening. ACE, angiotensin-converting enzyme inhibitor; ARB, angiotensin receptor blockers; CKD, chronic kidney disease; eGFR, estimated glomerular filtration rate

Figure 2 shows the results of one-way sensitivity analysis for 25% changes in parameter estimates on the ICER relative to the no screening scenario when using a risk threshold of 0.02 for the Bang et al. risk score with 2-year follow-up screening. Varying the costs of angiotensin-converting enzyme (ACE) inhibitors/angiotensin receptor blockers (ARBs) treatment had a large impact on the ICER relative to the no screening scenario. Increasing treatment cost parameters by 25% led to an ICER of $1,066,741 per QALY and decreasing treatment cost parameters by 25% resulted in cost savings. If costs are higher than the parameters used here, results will be very different.

**Fig. 3:**
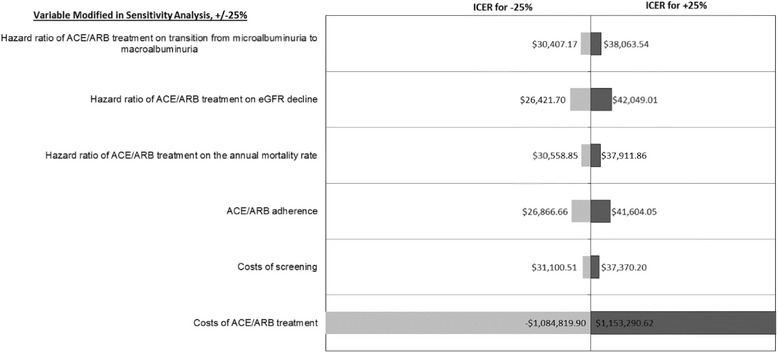
Sensitivity of results to 25% changes in specific parameters for screening and treatment, using a risk threshold of 0.05 for the Kshirsagar et al. risk score with 2-year follow-up screening relative to no screening. ACE, angiotensin-converting enzyme inhibitor; ARB, angiotensin receptor blockers; CKD, chronic kidney disease; eGFR, estimated glomerular filtration rate

Figure 3 shows a similar sensitivity analysis for the Kshirsagar et al. risk score using a risk threshold of 0.05 and 2-year follow-up screening. Similar to the Bang et al. risk score, only varying the costs of ACE/ARB treatment has a large impact on the ICER relative to the no screening scenario. Increasing treatment cost parameters by 25% led to an ICER of $1,045,704 per QALY and decreasing treatment cost parameters by 25% resulted in cost savings. If costs are higher than the parameters used here, results will be very different.

## Discussion

The Bang et al. and Kshirsagar et al. risk scores examined here, in general, produced a similar pattern of results. Using lower risk score thresholds when identifying persons for screening led to screening more people, identifying more early cases of moderate albuminuria, and saving more QALYs. However, because of costs associated with screening more persons, the incremental costs were greater for scenarios with lower risk score thresholds. Less frequent follow-up screening for person above the risk score threshold whose test was negative mitigated some of these additional costs while preserving most of the QALY gains from early detection. The ICER here summarizes the trade-off between increased QALYs and increased costs from broader screening. Importantly in these results, for 1-year follow-up screening, ICERs generally increased as risk thresholds decreased, whereas for 2-year and 5-year follow-up, ICERs decreased as risk thresholds decreased. This result illustrates that, with less frequent follow-up, few of the health gains of screening were lost while cost reductions were substantial.

The pattern of change in ICERs when moving to each lower risk score threshold was not always consistent due to different rates of change in the QALYs and costs at each threshold. Although costs and QALYs increased for all lower risk score thresholds, they did not always increase at the same rate. There were some specific differences in results between the two risk scores. The scenarios using the Kshirsagar et al. risk score to identify persons for screening produced slightly greater QALYs, but generally at higher costs than scenarios using the Bang et al. risk score at all thresholds. This could be because for each risk score threshold, the Kshirsagar et al. risk scores classifies more persons as high risk. This would lead to more persons screened and more QALYs gained, albeit at greater cost.

This study showed that there were several screening scenarios that were cost-effective for the given willingness to pay benchmark. Among all the cost-effective screening scenarios, the Kshirsagar et al. risk score with a threshold of 0.05 and 2-year follow-up screening and the Bang et al. risk score with threshold of 0.02 with 2-year follow-up screening generated the same maximum level of lifetime QALYs (21.373), but the one using the Bang et al. risk score had lower lifetime costs than that using Kshirsagar et al. risk score ($139,997 vs. $140,022) and can therefore be considered optimal. However, it should be noted that the difference in cost between these two screening scenarios is small ($25), so a clinician could optimally use either depending on the availability of patient data available to construct the alternative risk scores. It should also be noted that lifetime QALYs and costs had only small differences across all screening scenarios using both risk scores, so little costs or QALYs are gained incrementally, which should encourage caution when choosing a particular risk score threshold especially because the confidence intervals around lifetime costs and QALYs are relatively large.

For clinicians, this means, each patient could be evaluated using the Bang et al. risk score and screened for moderate albuminuria if the risk score is greater than 0.02. For example, persons older than age 50; those of any age with diabetes, hypertension, and anemia; or those of any age with a history of CVD would be candidates for CKD screening (Table 2). If the screening test is positive, the clinician would proceed with treatment; if negative, the clinician would conduct a follow-up screening in 2 years.

Past studies have found that screening the broad population for CKD may not be cost-effective, but screening populations at high risk, such as persons with diabetes or hypertension, may be cost-effective [12–14]. This study builds upon this past work by using risk scores to identify persons in the broader population to receive CKD screening. These risk scores rely not only on diabetes and hypertension, but also on age, gender, and health history, including CVD and anemia. Table 2 illustrates how this method of screening with risk scores leads to screening higher risk persons based on combinations of age, gender, CVD history, and anemia. These are persons that would not have been screened based on previous research showing that only screening those with diabetes or hypertension is cost-effective [12–14]. Using CKD risk scores allows for the examination of various thresholds for screening, which dichotomous criteria, such as history of diabetes or hypertension, does not. The information from this study could be used to frame future recommendations and programs for CKD screening that are not only effective from a clinical but also from a cost perspective.

This analysis is limited by the need to make assumptions regarding costs and other model parameters such as that all patients will present for initial and follow-up screening and be offered and accept treatment and that all providers will use risk scores and follow screening guidelines. The analysis only included medical costs and potentially omitted important societal costs, such as opportunity costs of time for screening, which would raise the ICERs associated with screening, and productivity losses and long-term care costs, which would decrease the ICERs. In addition, although model parameters were based on current epidemiologic literature, they may be imperfect or may omit additional unknown factors.

## Conclusions

In summary, the Bang et al. CKD risk score with a threshold of 0.02 and 2-year follow-up was found to be the most cost-effective for CKD screening. In contrast with current approaches for CKD screening that rely only on identifying high risk persons with diabetes or hypertension, CKD risk scores could be used by clinicians to identify a broader population for CKD screening. This is an important tool for increasing awareness of CKD and CKD screening in patients and clinicians. In particular, people with CKD who are detected in earlier stages of the disease would consequently benefit from receiving earlier clinical management and treatment to potentially slow down progression and prevent or delay ESRD.

## Abbreviations

ACE: Angiotensin-converting enzyme
ACR: Albumin-to-creatinine ratio
AHRQ: Agency for Healthcare Research and Quality
ARBs: Angiotensin receptor blockers
ARIC: Atherosclerosis Risk in Communities study
CHS: Cardiovascular Health Study
CKD: Chronic kidney disease
CMS: Centers for Medicare & Medicaid Services
CVD: Cardiovascular disease
eGFR: Estimated glomerular filtration rates
EHR: Electronic health record
Hb: Hemoglobin
ICER: Incremental cost-effectiveness ratios
NHANES: National Health and Nutrition Examination Survey
QALY: Quality adjusted life years
